# Heterogeneity and Development of Fine Astrocyte Morphology Captured by Diffraction-Limited Microscopy

**DOI:** 10.3389/fncel.2021.669280

**Published:** 2021-06-04

**Authors:** Daniel Minge, Cátia Domingos, Petr Unichenko, Charlotte Behringer, Alberto Pauletti, Stefanie Anders, Michel K. Herde, Andrea Delekate, Polina Gulakova, Susanne Schoch, Gabor C. Petzold, Christian Henneberger

**Affiliations:** ^1^Institute of Cellular Neurosciences, Medical Faculty, University of Bonn, Bonn, Germany; ^2^German Center for Neurodegenerative Diseases (DZNE), Bonn, Germany; ^3^Department of Neuropathology, University Hospital Bonn, Bonn, Germany; ^4^Division of Vascular Neurology, University Hospital Bonn, Bonn, Germany; ^5^Institute of Neurology, University College London, London, United Kingdom

**Keywords:** astrocytes, morphology, perisynaptic processes of astrocytes, microscopy, analysis, development, heterogeneity

## Abstract

The fine processes of single astrocytes can contact many thousands of synapses whose function they can modulate through bi-directional signaling. The spatial arrangement of astrocytic processes and neuronal structures is relevant for such interactions and for the support of neuronal signaling by astrocytes. At the same time, the geometry of perisynaptic astrocyte processes is variable and dynamically regulated. Studying these fine astrocyte processes represents a technical challenge, because many of them cannot be fully resolved by diffraction-limited microscopy. Therefore, we have established two indirect parameters of astrocyte morphology, which, while not fully resolving local geometry by design, provide statistical measures of astrocyte morphology: the fraction of tissue volume that astrocytes occupy and the density of resolvable astrocytic processes. Both are straightforward to obtain using widely available microscopy techniques. We here present the approach and demonstrate its robustness across various experimental conditions using mainly two-photon excitation fluorescence microscopy in acute slices and *in vivo* as well as modeling. Using these indirect measures allowed us to analyze the morphology of relatively large populations of astrocytes. Doing so we captured the heterogeneity of astrocytes within and between the layers of the hippocampal CA1 region and the developmental profile of astrocyte morphology. This demonstrates that volume fraction (VF) and segment density are useful parameters for describing the structure of astrocytes. They are also suitable for online monitoring of astrocyte morphology with widely available microscopy techniques.

## Introduction

Astrocytes have several distinct morphological features that set them apart from other brain cells. For instance, individual astrocytes and their processes occupy territories that largely do not overlap between neighbors (Bushong et al., 2002). Within those territories, their processes branch out extensively forming the so-called gliapil, into which many thousand synapses can be embedded. The processes that approach synapses are called perisynaptic astrocyte processes, which can be very thin (~100–200 nm; Ventura and Harris, 1999; Witcher et al., 2007). Because of their proximity to synapses, they are believed to be important sites for neuron-astrocyte interactions. However, not all synapses are necessarily approached by fine astrocytic processes and, if they are, the contact area can be highly variable. In the hippocampus, for instance, only ~40–60% of synapses are directly apposed by astrocyte processes (Ventura and Harris, 1999; Witcher et al., 2007). This variability has functional consequences for synapse function. In the supraoptic nucleus, for example, a reduction of the astrocytic coverage of neurons leads to a decreased uptake of synaptically released glutamate, which increases the activation of presynaptic glutamate receptors (Oliet et al., 2001). In the hippocampus, we could recently demonstrate that the relative coverage of spines by astrocytes depends on the spine size and sets the local efficacy of glutamate uptake (Herde et al., 2020). Importantly, the spatial arrangement of astrocyte processes and synapses is not static. For instance, astrocytic processes have been demonstrated to be motile in the brainstem and hippocampus (Hirrlinger et al., 2004; Haber et al., 2006), and especially plasticity-inducing stimuli are potent triggers of changes of perisynaptic process morphology (Genoud et al., 2006; Lushnikova et al., 2009; Bernardinelli et al., 2014; Henneberger et al., 2020), which can alter synapse stability and the spatial precision of synaptic transmission (Bernardinelli et al., 2014; Henneberger et al., 2020). In addition, it is well-established that pathophysiological conditions can lead to strong astrocyte morphology changes. Therefore, revealing how the morphology of these small astrocyte processes and their dynamic changes shape synapse behavior is important for understanding brain function.

However, this remains technically challenging because a substantial proportion of astrocytic processes are too small to be fully resolved by widely available diffraction-limited microscopy, for review see Heller and Rusakov (2017). There are of course techniques that have the necessary higher spatial resolution (Heller and Rusakov, 2017), but each has its specific advantages and disadvantages. Electron microscopy, for instance, has provided many important insights into astrocyte morphology, into structural synapse-astrocyte relationships and their potential functional implications (Ventura and Harris, 1999; Genoud et al., 2006; Witcher et al., 2007; Lushnikova et al., 2009; Patrushev et al., 2013; Medvedev et al., 2014). One caveat of electron microscopy and many other suitable super-resolution techniques is that they require chemical tissue fixation or have been used successfully mostly in fixed tissue, for review see (Heller and Rusakov, 2017). There are two disadvantages of using fixed tissue. First, fixation can potentially lead to artifacts depending on the fixation protocol as demonstrated for astrocytes (Korogod et al., 2015). Second, it is incompatible with real-time monitoring of astrocytic morphology changes. These obstacles can be overcome by stimulated-emission depletion (STED) imaging, which has been successfully used to visualize astrocytes at the necessary resolution in living brain tissue (Tønnesen et al., 2018; Arizono et al., 2020, 2021; Henneberger et al., 2020). However, three-dimensional STED imaging typically requires high illumination powers, which can limit the number of image acquisitions, and is at least currently not widely available.

Therefore, we previously started to investigate whether information about small astrocytic processes can be extracted from data obtained by diffraction-limited microscopy from organized tissue, i.e., from tissue in which the three-dimensional structure of astrocytes is preserved. We have previously demonstrated that measuring the fraction of tissue volume occupied by astrocytes can be used to obtain information about the local astrocyte structure (Medvedev et al., 2014; King et al., 2020) and to detect changes of perisynaptic astrocyte processes (Henneberger et al., 2020). Here, we present an additional indirect measure of astrocyte structure, the segment density, and support the usefulness of both parameters with simulations and experiments. Since both parameters are easily computed, they allowed us to study relatively large populations of astrocytes *in situ*. Analyzing several hundred astrocytes, we found that the fraction of tissue volume occupied by astrocytes varies between regions of the hippocampus but is surprisingly constant during a rodent’s life. In contrast, the complexity of the branching pattern, captured by the segment density, depended on the hippocampal subregion and developmental stage.

## Materials and Methods

### Animals

Transgenic mice expressing EGFP under a GFAP promotor (Nolte et al., 2001) of either gender were used throughout this study (FVB background). Their age ranged from 7 to 570 days and is indicated for individual experiments. Animals were housed under 12 h light/dark conditions with food and water *ad libitum*. All animal procedures were conducted in accordance with the regulations of the European Commission and all relevant national and institutional guidelines and requirements. All procedures have been approved by the Landesamt für Natur, Umwelt und Verbraucherschutz Nordrhein-Westfalen (LANUV, Germany) where required.

### Virus Injections

In a subset of experiments, tdTomato was expressed in hippocampal astrocytes using recombinant adeno-associated viruses (rAAV). tdTomato expression was achieved by replacing iGluSnFR (Marvin et al., 2013) from pAAV1.GFAP.iGluSnFr.WPRE.SV40 (plasmid obtained from PennCore, addgene #98930) with tdTomato (addgene #62726) *via* BamHI/HindIII restriction sites. The resulting plasmid, pAAV1.GFAP.tdTomato.WPRE.SV40, was verified by sequencing. AAV1.GFAP.tdTomato.WPRE.SV40 viruses (serotype 1/2) were produced in HEK293T cells, harvested and purified as described before (Woitecki et al., 2016). Briefly, the AAV1.GFAP.tdTomato.WPRE.SV40 plasmid was co-expressed with the helper plasmids pRV1, pH21 and pFΔ6 in HEK283T cells and harvested ~48 h later. Next, cells were lysed, virus particles were purified by HiTrapTN heparin columns and concentrated with Amicon Ultra Centrifuge Filters to a final stock of 500 μl. Intrahippocampal virus injections were administered as described before (Herde et al., 2020). Briefly, 4–5-week-old mice were anesthetized through intraperitoneal (i.p.) injection of Fentanyl/Midazolam/Medetomidin (0.05/5.0/0.5 mg/kg body weight) and stereotactically injected (coordinates for the dorsal hippocampus, relative to bregma: anterior −1.8 mm, lateral +/− 1.6 mm, ventral −1.6 mm) with 1 μl of virus AAV1/2.GFAP.tdTomato (virus titer: ~10^9^ viral particles per μL, injection speed: 100 nl/min). anesthesia was terminated by Naloxon/Flumazenil/Atipamezol (1.2/0.5/2.5 mg/kg body weight, i.p. injection). Animals were sacrificed 2–4 weeks after virus injection.

### Preparation of Acute Hippocampal Slices

Acute slices were prepared from mice as previously described (Anders et al., 2014; Minge et al., 2017). Briefly, mice between postnatal day 7 and 570 were anesthetized with isoflurane and decapitated. 300 μm thick slices from the dorsal hippocampus (coronal), ventral hippocampus (horizontal) or somatosensory cortex (horizontal, layer 2/3) were obtained using a vibratome (Campden Instruments, UK) in an ice-cold slicing solution containing (in mM): NaCl 60, sucrose 105, KCl 2.5, MgCl_2_ 7, NaH_2_PO_4_ 1.25, ascorbic acid 1.3, sodium pyruvate 3, NaHCO_3_ 26, CaCl_2_ 0.5, and glucose 10 (osmolality 300–310 mOsm/kg), and kept in the slicing solution at 34°C for 15 min before being stored at room temperature in an extracellular solution containing (in mM): NaCl 126, KCl 2.5, MgSO_4_ 1.3, NaH_2_PO_4_ 1.25, NaHCO_3_ 26, CaCl_2_ 2, and glucose 10 (osmolality 297–303 mOsm/kg). Slices were allowed to rest for at least 45 min before the experiments, which were performed at 33–34°C in a submerged recording chamber. All solutions were constantly bubbled with 95% O_2_/5% CO_2_. In some experiments, the osmolarity of the extracellular solution was rapidly changed to ~200 mOsm/kg or ~400 mOsm/kg. This was achieved by switching to an extracellular solution with a reduced concentration of NaCl (61 mM) or one to which sucrose had been added (100 mM). In these experiments, a baseline image of an EGFP-expressing astrocyte was taken (see below) before the solution exchange and then another one 10 min after the solution exchange had begun.

### Two-Photon Excitation (2PE) Fluorescence Microscopy in Acute Slices

Astrocytes expressing fluorescent proteins or filled with fluorescent dyes were visualized using a Scientifica 2PE fluorescence microscope with a 40× /0.8 NA objective (Olympus) or an Olympus FV10MP 2PE fluorescence microscope with a 25× /1.05 NA objective and a pulsed infrared laser (Vision S, Coherent, *λ* = 800 nm unless stated otherwise). Laser power was adjusted for depth to obtain a fluorescence intensity equivalent to that recorded with a laser power of 2–3 mW at the slice surface. In a subset of experiments, EGFP-expressing astrocytes were dialyzed for 20 min with the gap junction impermeable red fluorescent dye Texas Red dextran 3 kDa (300 μM; Thermo Fisher Scientific, USA) *via* standard patch-pipettes (3.5–4.5 MΩ) filled with an intracellular solution containing (in mM): KCH_3_O_3_S 135, HEPES 10, di-Tris-Phosphocreatine 10, MgCl_2_ 4, Na_2_-ATP 4, Na-GTP 0.4 (pH adjusted to 7.2 using KOH, osmolarity 290–295 mOsm/kg). For further details see Henneberger et al. (2010) and Henneberger and Rusakov (2012). The access resistance was continuously monitored, and recordings were rejected if theyt exceeded 30 MΩ at the beginning of the recording or changed by more than 30% during dye-loading (20 min). Images and image stacks of astrocytes were routinely acquired with a nominal resolution of 0.04–0.09 μm/pixel in the x-y plane (512 × 512–2048 × 2048 pixels) and in 1 μm steps in the z-direction. The cell bodies of all studied astrocytes were >80 μm below the tissue surface.

### *In vivo* Two-Photon Excitation (2PE) Fluorescence Microscopy

Acute *in vivo* imaging experiments were performed in 8–12-week-old animals as described previously (Monai et al., 2016; King et al., 2020) using isoflurane inhalation anesthesia (3% for induction, 1.3–1.5% for maintenance) and additional buprenorphine analgesia (0.1 mg/kg). Briefly, the mouse head was fixed in a stereotaxic apparatus, the skull was exposed and a small craniotomy (about 3 mm diameter, 1.5 mm posterior to bregma and 3.5 mm lateral from the midline) above the right barrel cortex was performed using a dental drill. The skull, but not the dura mater, was carefully removed. The cortex was covered with a 1.2% low-melting agarose. A glass coverslip (4 mm in diameter, Warner Instruments, Hamden, USA) was placed on top and secured by dental cement (Temdent Classic, Schütz Dental GmbH, Rosbach, Germany). A custom-made metal frame was attached to the skull using dental cement. Eyes were covered with bepanthen (Bayer Vital GmbH, Leverkusen, Germany) during the whole surgical and imaging procedure. Recordings were then performed under continued isoflurane inhalation anesthesia (1.2–1.5%), for which mice were head-fixed to another stereotaxic frame and placed under a 2PE microscope (COSYS Ltd, East Sussex, UK) equipped with a femtosecond infrared pulsed Mai Tai HP laser (Spectra-Physics, wavelength set to 800 nm) and a 16× water-immersion objective lens (Nikon LWD, NA 0.8). To avoid brain tissue damage, the laser power did not exceed 30 mW at the front lens of the objective. Images (1024 × 1024 pixels) were acquired at a depth of 100–150 μm from the pia with a nominal resolution of ~0.100 μm/pixel using ScanImage 5.6. Animals were kept at a constant temperature of 37°C throughout surgery and the experiment by placing them on a heating blanket. Data were analyzed offline as explained below.

### Expansion Microscopy (ExM)

Expansion Microscopy (ExM; Chen et al., 2015; Chozinski et al., 2016) of EGFP-expressing astrocytes was performed as previously described (Deshpande et al., 2017; Bürgers et al., 2019; Herde et al., 2020). Briefly, 4–10-week-old animals were deeply anesthetized and transcardially perfused with 4% paraformaldehyde (PFA) in phosphate-buffered saline (PBS, pH 7.4). Their brains were removed and fixed at 4°C either overnight (ON) or for 1–2 h. Coronal sections of 70 μm thickness were cut on a vibratome and incubated in permeabilization buffer (0.5% Triton X-100 in PBS pH 7.4, ON, 4°C). Slices were incubated with a chicken anti GFP (1:5,000; Abcam ab13970, lot GR89472–16) primary antibody (24 or 48 h, 4°C). After 3 × 20 min washing in permeabilization buffer, slices were incubated with a secondary antibody (anti-chicken Alexa Fluor 488, 1:200; ThermoFisher A11039, 4°C, 24 h). After washing in PBS, slices were incubated with Hoechst 33342 (1:2,000, Invitrogen H3570, lot 1874027) in distilled water for 10 min at RT. After washing again, slices were imaged in PBS containing 0.05% p-phenylenediamine before expansion with a 20× /0.75 NA or a 40× /1.1 NA objective in a Leica SP8 confocal microscope. Further treatment was adopted from Chen et al. (2015), Chozinski et al. (2016) and similar to Herde et al. (2020). In short, slices were incubated in 1 mM methylacrylic acid-NHS (Sigma Aldrich #730300, RT, 1 h). After 3 × 20 min washing in PBS, slices were incubated for 45 min in monomer solution (in g/100 ml PBS: 8.6 sodium acrylate, 2.5 acrylamide, 0.15 N,N′-methylenebisacrylamide, 11.7 NaCl, 4°C). Slices were then incubated with gelling solution (monomer solution supplemented with %(w/v): 0.01 4-hydroxy-TEMPO, 0.2 TEMED, 0.2 APS) at 4 °C for 5 min before being transferred to a chamber sandwiched between two coverslips at 37°C for 2 h. Coverslips were removed and proteins were digested in digestion buffer (50 mm Tris pH 8, 1 mm EDTA, 0.5% Triton X-100, 0.8 M guanidine, 16 U/ml proteinase K) at 37°C for 12–14 h. For expansion, slices were incubated for 2.5 h in distilled water at RT and water was exchanged every 15–20 min. Finally, slices were mounted on poly-lysine coated m-Slide 2 well Ibidi-chambers and sealed with a poly-lysine coated coverslip on top, adding a drop of water to prevent the gel from drying. All imaging was performed on a Leica SP8 confocal microscope using a 40× /1.1 NA objective and hybrid detectors. For each sample, the expansion factor (~4–5 over all experiments) was determined by identifying the same cells labeled with Hoechst 33342 in the dentate gyrus before and after expansion and then measuring their sizes pre- and post-expansion. For analysis, image stacks of EGFP-expressing astrocytes were acquired (x-y-z, typically ~2,500 × 2,500 × 15 voxels, voxel dimensions ~0.1 μm × 0.1 μm × 0.4 μm, corresponding to pre-expansion dimensions of ~0.025 μm × 0.025 μm × 0.10 μm). Images were then processed using Huygens Essential and analyzed as explained below.

### Image Analysis and Quantification of Volume Fraction and Segment Density

Image data were processed using in-house written scripts in MATLAB (Mathworks). All images of astrocytes had a nominal resolution of at least 0.1 μm/pixel and contained a single optical section through the center of the astrocyte soma. Before further analysis, the background intensity was determined in a region without any labeled structures and subtracted from all pixels. All images were then processed using a median filter (0.3 μm × 0.3 μm). For experimental data, a circular region of interest (ROI) was placed at the center of the soma for obtaining the fluorescence intensity corresponding to a volume fraction (VF) of 100% (reference ROI, see for instance Figure 3). A second analysis ROI was then defined, in which the astrocytic VF was determined by taking the average fluorescence intensity in this ROI and dividing it by the value corresponding to 100%. For analyzing the distribution of the astrocytic VF (Figure 3D), the analysis ROI was cut into quadratic subregions and the VF for each subregion was determined. Please see the next section for simulated data and their analysis.

**Figure 1:**
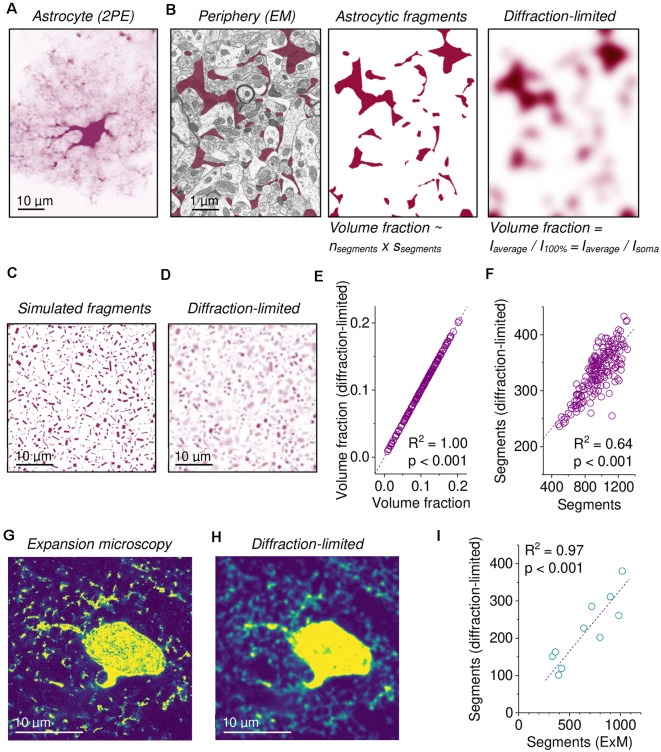
Assessing fine astroglial morphology in diffraction-limited microscopy by measuring astroglial volume fraction (VF) and segment density. **(A)** Example of a single EGFP-expressing astrocyte imaged using diffraction-limited two-photon excitation (2PE) fluorescence microscopy (single focal plane through the soma). Note the blurry periphery representing small astroglial processes. **(B)** Left panel: example of an electron micrograph of the hippocampal neuropil with clearly delineated astrocyte fragments highlighted in purple (left panel). For further experimental details see Medvedev et al. (2014). Middle panel: same section only showing astrocyte fragments. The fraction of the section that is occupied by astroglial segments is the product of their number (n_segments_) and average size (s_segments_) divided by the total area. Right panel: diffraction-limited microscopy was illustrated by applying a Gaussian filter with a FWHM of 500 nm. Astrocyte fragments cannot be clearly separated and counted. The fraction of occupied area/volume can be calculated by normalizing the average intensity (I_average_) to the value corresponding to 100% (I_100%_). **(C)** An example of a single section at full resolution cutting through simulated astrocyte processes of various sizes and orientations in a simulated image stack (see text and methods). **(D)** Emulation of diffraction-limited fluorescence imaging of the image stack from **(C)**. Note that this is not a smoothed version of **(C)** but instead representative of diffraction-limited imaging of the entire stack. Also, note the now blurry and overlapping astrocyte processes. **(E)** The VF obtained from simulated diffraction-limited microscopy (I_average_/I_100%_) strongly depends on the original fraction of volume occupied by astrocyte processes (linear fit, *n* = 190 separate sets of astrocyte processes). **(F)** The number of detectable astrocyte segments was determined (see text and methods) in simulations of diffraction-limited microscopy **(D)**. The number of detected segments was smaller than the number of simulated astrocyte processes but showed a highly significant positive correlation (linear fit, same data set as in **(E)**. **(G)** Example of super-resolution expansion microscopy (ExM) of an EGFP-expressing astrocyte (single focal plane from a stack). Note the high level of detail. **(H)** Simulation of diffraction-limited microscopy of ExM data (same cell as in **G**). Note the appearance of out-of-focus structures and blurring. **(I)** The number of astrocyte segments determined in ExM was lower after simulation of diffraction-limited imaging but strongly correlated with the number obtained directly from ExM data (linear fit, *n* = 10 independent experiments).

**Figure 2:**
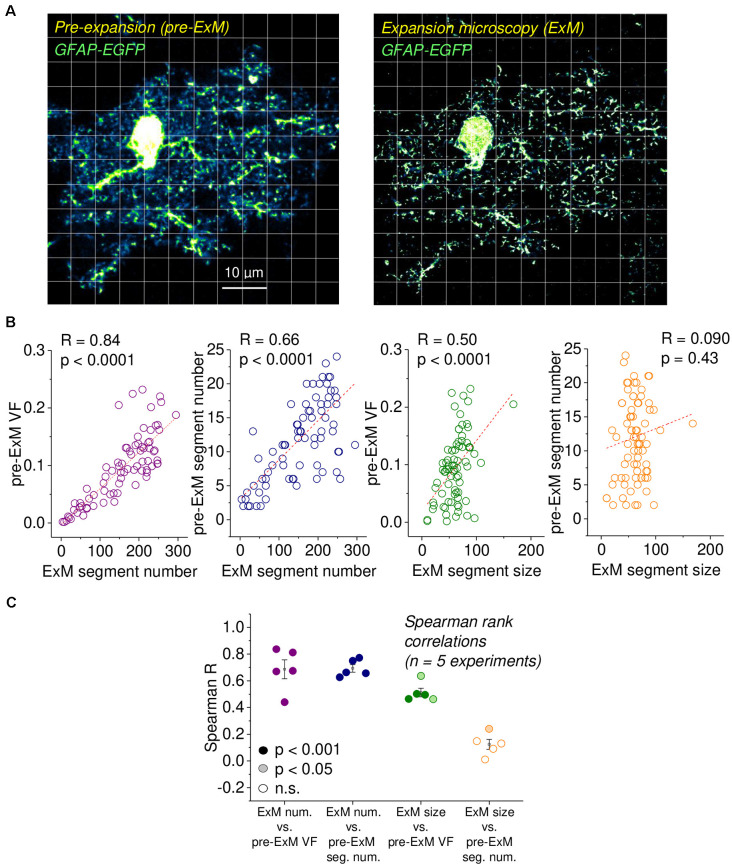
Validation of astrocytic volume fraction (VF) and segment density using expansion microscopy (ExM). **(A)** Example of an EGFP-expressing astrocyte before expansion (left panel, pre-ExM) and after expansion (right panel, ExM), both obtained using confocal microscopy. The ExM image was rescaled to the original size of the tissue. The scale bar applies to both panels and refers to the pre-ExM dimension. Note the highly resolved small astrocyte processes and the disappearance of out-of-focus structures in ExM. The grid illustrates the regions of interest (ROIs) for both images. The soma and areas without astrocyte structures were excluded from the analysis. Experimental data from Herde et al. (2020). **(B)** For each ROI in **(A)**, the VF and segment number were determined from the pre-ExM image and compared with the average segment size and number of segments measured in the ExM image in the same ROI. Strong correlations were found between the astrocyte segment number after expansion with the pre-expansion VF and segment number (1st and 2nd panel from the left) and between the astrocytic segment size after ExM and the pre-expansion VF (3rd panel from the left) but not between the astrocytic segment size after ExM and the pre-expansion segment number. Spearman rank correlations throughout (*n* = 77 ROIs). Dashed lines are linear fits, only for illustration. **(C)** Spearman’s rank correlation coefficients for five independent experiments, as illustrated in **(A,B)**. Filling represents statistical significance (dark for *p* < 0.001, light for *p* < 0.05 and white for *p* > 0.20).

**Figure 3:**
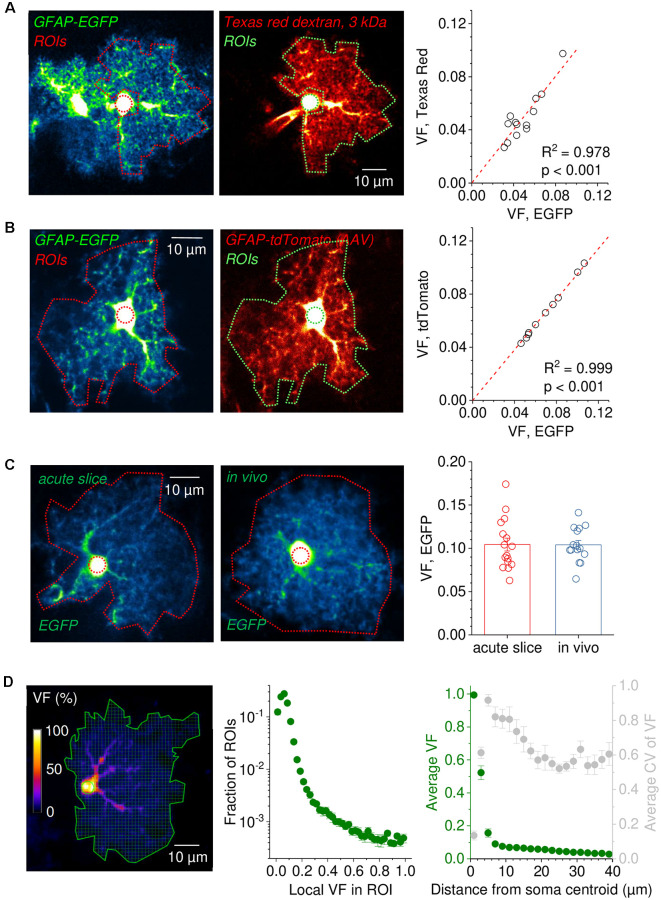
Robustness of astrocytic volume fraction (VF) measurements across experimental conditions. **(A)** Example of an EGFP-expressing astrocyte before (left panel) and after patching and filling with the gap junction impermeable dye Texas Red dextran 3 kDa for 20 min (middle panel, patch pipette visible left of the soma). Note the similar appearance of the patched cell and the previous EGFP fluorescence). The somatic reference regions of interest (ROIs) are illustrated by a circular dotted line. ROIs for analysis sparing the soma and perisomatic region are also delineated by dotted lines. Pseudo color images for revealing low-intensity details. Right panel: Comparison of volume fractions (VF) obtained by normalizing the average fluorescence in the analysis ROI to that in the somatic reference ROI. The VFs of EGFP-expressing cells are tightly correlated with the VFs obtained after filling the same cells with Texas Red dextran 3 kDa (linear regression through origin, *n* = 13). **(B)** Analysis of astrocytes simultaneously expressing the two fluorescent proteins EGFP and tdTomato. An example of an astrocyte expressing both EGFP and tdTomato is shown in the left (EGFP) and middle panel (tdTomato). Dotted lines represent the analysis and somatic reference ROIs. VF values obtained from analysis of the EGFP and tdTomato fluorescence using the same ROIs were virtually identical (linear regression through origin, *n* = 10, right panel). **(C)** Comparison of VFs of EGFP-expressing astrocytes in the somatosensory cortex (layer 2/3) between horizontal slice preparations and in anesthetized mice* in vivo*. Example images of EGFP-expressing astrocytes in an acute slice (left) and *in vivo* (middle). Dotted lines delineate the ROI for analysis including the soma (outer) and the somatic reference ROI (inner, circular). On average, VFs from both preparations were nearly identical (unpaired Student’s *t*-test: *p* = 0.966, *n* = 16 astrocytes in slices from two independent preparations, *n* = 15 astrocytes *in vivo* from three different animals). **(D)** Analysis of VF distribution within the territory of a single astrocyte. The entire territory (solid green line) of a sample EGFP-expressing astrocyte is divided into ROIs of ~1 × 1 μm^2^ (left panel). The fluorescence intensity of each ROI is normalized to the somatic reference ROI (solid green circle) and the VF distribution across ROIs is calculated. Middle panel: average VFs distribution of hippocampal astrocytes [CA1, stratum radiatum (SR), *n* = 10]. Right panel: the dependence of the VF on the distance from the soma was calculated by averaging the VF of all ROIs at the given distances in each cell and then averaging the obtained data across all cells (same data set, green, left axis). The average coefficient of variation of VF is also plotted against distance from the soma (gray, right axis). In panels **(A–C)**, the contrast of example images has been enhanced for display purposes and the scale bars apply to both panels.

To obtain a measure related to the number of astrocytic processes in the analysis ROI, the image was then binarized using the fluorescence intensity corresponding to the VF as a threshold. All continuous objects in the binarized image with a size of more than one pixel were considered to be astrocytic segments and counted. For experimental data, the number of segments was normalized to the area of the analysis ROI (segment density in μm^−2^), which is more useful for comparison between astrocytes with varying territory sizes.

**Figure 4:**
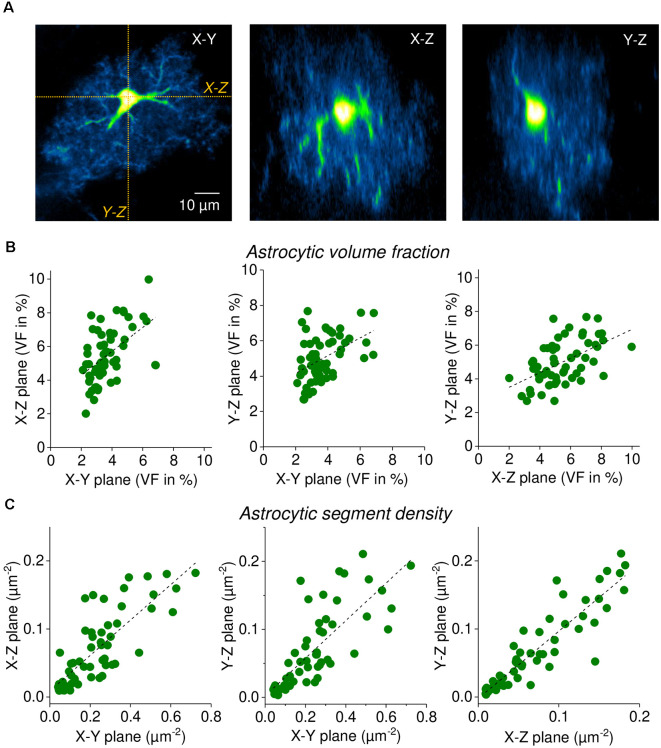
Single focal plane measurements of astrocytic volume fraction (VF) and segment density are representative of three-dimensional morphology. **(A)** Sample EGFP-expressing astrocyte (CA1 stratum radiatum, acute slice). Left panel: single horizontal focal plane (X-Y) through the soma from an image stack (2PE fluorescence microscopy, nominal x-y resolution of 0.09 μm/pixel, z-steps of 1 μm). X-Z and Y-Z sections through the stack were taken as indicated (orange dotted lines) and labeled. Middle panel: X-Z section (same scale as in left panel). Right panel: Y-Z section (same scale as in left panel). Note the reduced resolution along the Z-axis. In all three planes, the astrocytic volume fraction and segment density was determined (*n* = 58 astrocytes). **(B)** Correlation of astrocytic volume fractions (in %) between planes. Spearman’s rank correlations from left to right: *R* = 0.520, *p* < 0.0001; *R* = 0.422, *p* = 0.000976; *R* = 0.552, *p* < 0.0001. Linear fits as a visual guide (dashed black lines). **(C)** Correlation of astrocytic segment densities (in segments per μm^2^) between planes. Spearman’s rank correlations from left to right: *R* = 0.812, *p* < 0.0001; *R* = 0.931, *p* < 0.0001; *R* = 0.859, *p* < 0.0001. Linear fits as a visual guide (dashed black lines). Note that segment densities are generally lower in planes along the Z-axis because of the reduced resolution (see text). In these analyses, large processes were excluded from the analysis to focus on fine astrocyte processes.

In a subset of experiments (Figure 4), image stacks of astrocytes were analyzed to compare measurements of astrocytic VF and segment density along image planes in x-y, x-z and y-z orientations. Here, the image stacks were resliced as indicated in Figure 4 and each section was analyzed as described above.

For the analysis of data obtained from ExM (see above) for Figure 1, the astrocytic VF and segment density were first determined from the central slice of the original data stack, as explained above. The ExM image stack was then convolved with a point spread function [PSF, full width at half-maximum (FWHM) of 0.45 μm in x-y and of 0.90 μm in z] to emulate diffraction-limited microscopy. The astrocytic VF and segment density were then determined a second time from the same central slice of the convolved image stack with identical reference and analysis ROIs. For the comparison of VF and segment densities measured before expansion with ExM data (Figure 2), images of astrocytes taken before and after expansion (pre-ExM and ExM respectively) were aligned and divided into identical rectangular ROIs. For each cell, all ROIs except somatic ROIs and ROIs without astrocyte structures were analyzed separately. For pre-ExM images, the VF and segment number were determined for each ROI as explained above. For ExM images, the number and average size of astrocyte segments in each ROI were determined from images that had been binarized as explained above.

### Simulations of Diffraction-Limited Microscopy of Astroglial Processes

An optical section through an astrocyte contains cross-sections of astrocyte processes with sizes that are typically close to or below the diffraction limit of 2PE fluorescence microscopy. We established the relationship between the number and size of astrocyte processes and astrocyte volume fraction and segment density (see above) by mimicking a typical experiment. Non-overlapping virtual astrocyte processes were approximated by rectangular cuboids and placed randomly in a 3D tissue block (matrix of 2,000 × 2,000 × 31 voxels corresponding to 40.0 μm × 40.0 μm × 3.1 μm, all voxels initialized to 0) by setting all voxels within all cuboids to a value of 255. A set of these 3D tissue blocks filled with astrocyte processes was generated by randomly varying the numbers and sizes of simulated astrocyte processes (Visual Studio C++ and OpenCV image processing library). The generated tissue blocks were then convolved with a three-dimensional Gaussian PSF with an FWHM of 0.45 μm in the x-y plane and 0.90 μm in the z-direction to simulate diffraction-limited imaging (MATLAB, Mathworks). The central x-y plane of the convolved matrix is equivalent to a single focal section using diffraction-limited imaging and was further analyzed. First, the volume fraction was calculated by taking the average voxel intensity and normalizing it to the value corresponding to 100% (255, see above). Second, the number of segments was determined as described above. Both were then correlated with the number of astrocyte processes that were initially placed in the simulated 3D tissue block and the known fraction of volume occupied by virtual astrocyte processes in the 3D tissue block.

### Quantification and Statistical Analysis

Image analysis was performed in FIJI/ImageJ (NIH) and MATLAB (Mathworks). Numerical and statistical analyses were performed in Excel (Microsoft), Origin Pro (OriginLab Corporation) and MATLAB (Mathworks). In the text, results are given as mean ± s.e.m. unless stated otherwise. n denotes the number of experiments. In graphs, statistical significance is indicated by asterisks. * for *p* < 0.05, ** for *p* < 0.01 and *** for *p* < 0.001. Statistical tests such as Student’s *t*-tests, ANOVA, Kruskal-Wallis test and Spearman’s rank correlations were always two-tailed and used as appropriate and as indicated in the text and figure legends. The Shapiro-Wilk test was used to establish if data followed a normal distribution.

## Results

### Parameters of Fine Astrocyte Morphology in Diffraction-Limited Microscopy

Diffraction-limited microscopy cannot fully resolve the complex morphology of the fine peripheral processes of astrocytes (see “Introduction” section) and how these processes are precisely arranged around synapses, for instance. Nonetheless, it captures the distribution of labeled astrocyte structures. A typical example of an astrocyte expressing EGFP in its cytosol (Figure 1A) illustrates how large structures with high volume such as the soma or big branches are represented by a high fluorescence intensity whereas the fine branches in the periphery are relatively dim and blurry. To illustrate how the latter arises from astrocytic structures we re-investigated electron microscopic sections in which astrocytic structures had been identified, published data from Medvedev et al. (2014) and Henneberger et al. (2020) (Figure 1B, left panel). Looking only at the cross-sections of astrocytic processes in this example, called segments in the following (Figure 1B, middle panel), this tissue section can be characterized by the number of astrocytic segments (n_segments_) and their average size (s_segments_). The product of both divided by the total area of the tissue section is equal to the area fraction, i.e., the fraction of area occupied by astrocytic segments. When the thickness of the tissue section is also considered, the corresponding measure is the astrocyte volume fraction, i.e., the fraction of volume of the tissue section occupied by astrocyte processes. Importantly, the area fraction is a consistent estimate of the volume fraction, which is known as the Delesse principle, also see Chayes (1953). Thus, the astrocyte volume fraction is an indirect measure of astrocyte process number and their average size in a tissue section. It is also an indicator of dynamic changes because it increases when astrocyte processes become more abundant or bigger, for instance. When diffraction-limited microscopy of the same section is simulated by convolving the image with a typical point spread function (PSF), a blurry image reminiscent of the periphery of the astrocyte in Figure 1A is obtained and astrocyte segments cannot be clearly identified any longer (Figure 1B, right panel). Therefore, the volume fraction needs to be extracted by different means. If all astrocyte processes in a tissue section are equally filled with a dye/label, then the fraction of volume they occupy is given by the cumulative intensity of that label divided by the value corresponding to the tissue section being entirely filled by astrocytic structures. When divided by the number of pixels/voxels of the tissue sections, this corresponds to the average label intensity I_average_ divided by I_100%_. One area, for which 100% occupancy by astrocytes can be safely assumed, is the somatic region because the soma is much larger than a typical PSF (Medvedev et al., 2014; Savtchenko et al., 2018; Henneberger et al., 2020). This procedure requires that pixels/voxels devoid of astrocyte processes have a fluorescence intensity of zero. Therefore, background fluorescence and other offsets need to be carefully subtracted before analysis. Thus, the astrocytic volume fraction VF can be calculated from diffraction-limited microscopy as a measure of both the number and size of astrocytic processes in a region of interest. Importantly, we have previously shown that this VF measure from diffraction-limited microscopy matches that obtained by electron microscopy (Medvedev et al., 2014).

In addition, we established a second measure of astrocyte morphology that is correlated with the number of astrocytic processes in the analyzed tissue section. It is obtained by binarizing the image using a fluorescence intensity threshold. This threshold is set to the fluorescence intensity that is equivalent to the volume fraction in the analyzed ROI, which is I_average_ (Figure 1B, right panel). This threshold was chosen because it does not require any manual adjustments by the investigator and is instead fully determined by the analyzed image itself. Then the number of confluent supra-threshold areas is determined (number of segments, also see “Material and Methods” section). It should be noted that this approach links the threshold for segment detection to the volume fraction. Alternatively, the threshold could be set to a fixed percentage of the somatic fluorescence intensity.

We next quantified the extent to which the astrocyte VF and the number of segments reveal details of astrocyte morphology using simulations. Diffraction-limited imaging of astrocytic processes was emulated by randomly placing cuboids of varying geometry and number into simulated three-dimensional tissue sections, which were then convolved by a PSF typical for our imaging conditions (Figures 1C,D). For each simulated tissue section, the VF and number of segments were determined as described above and correlated with the fraction of simulated voxels filled by cuboids and the number of simulated cuboids, respectively (Figures 1E,F). As expected, diffraction-limited microscopy does not affect the value of the VF (Figure 1E). The number of detected segments also displayed a highly significant but weaker correlation (Figure 1F). This number of detected segments in simulated diffraction-limited microscopy is lower than the simulated astrocytic cuboids (by a factor of two to three in these simulations), which is expected because of the limited spatial resolution of diffraction-limited microscopy.

To test this relationship on real astrocytes, we re-analyzed previously published superresolution fluorescence microscopy data obtained by expansion microscopy (ExM) of astrocytes expressing EGFP (Herde et al., 2020; Figure 1G). The spatial resolution of ExM in these experiments is increased by a factor of four to five compared to standard confocal microscopy. Similar to the simulated data above, we emulated diffraction-limited microscopy by convolving ExM image stacks with a typical PSF (Figure 1H). We then compared the number of detectable segments in ExM and in convolved ExM data using the procedure explained above (Figure 1I). Again, the number of segments was highly correlated, and emulating diffraction-limited microscopy reduced the number of detectable astrocyte processes in the image.

Next, we further explored how local characteristics of astrocyte structure are captured by the VF and segment number by re-analyzing a subset of experiments from Herde et al. (2020), in which we had managed to obtain confocal images of an EGFP-expressing astrocyte before expansion and after expansion of the tissue sample (pre-ExM and ExM, respectively; Figure 2A). The higher spatial resolution of ExM allowed us to obtain more precise measurements of the number of astrocyte segments and their average size in for each ROI (illustrated by the grid in Figure 2A), which we then compared to the VF and segment number measured from diffraction-limited fluorescence imaging (pre-ExM) in the same ROI as explained above. The prediction was that if a ROI contained a high number of astrocyte segments in ExM, then the VF and number of segments obtained in that ROI in diffraction-limited microscopy should both be high too. In addition, if the average size of astrocyte segments in ExM is high in an ROI, then the VF in the ROI in the pre-ExM data should be high too. Indeed, this was observed for the example cell (Figures 2A,B) and across a total of five astrocytes (Figure 2C). In contrast, the size of astrocyte segments in ExM and the number of detected segments before the expansion was only weakly correlated in one out of five cells (Figure 2C, left panel).

Overall, these experiments demonstrate that the volume fraction and the number of segments can be easily extracted from imaging data and that both are indirect but reliable measures of the number and size of fine astrocyte processes.

### Probing the Reliability of Volume Fraction Measurements

Volume fraction (VF) measurements need to be consistent across experimental conditions to be widely applicable. We, therefore, tested the robustness of VF using several approaches. First, we reasoned that if fluorescent dyes fulfil the important requirement of having a constant concentration throughout the astrocyte cytosol then they should report the same VF regardless of the specific properties of the dye. This was tested in acute hippocampal slices with astrocytes expressing EGFP. In a first set of experiments, single EGFP-expressing cells were selected, and an image was taken (Figure 3A, left panel). Cells were then filled with dextran conjugated Texas Red (3 kDa), which cannot pass through gap junctions because of its molecular weight (Figure 3A, middle panel), and another image was taken. A somatic reference ROI and an analysis ROI covering the astrocyte territory (sparing the soma and the perisomatic region) were defined and the VF was determined from both images with identical ROIs. The analysis revealed that both VFs were highly correlated (Figure 3A, right panel, linear fit through the origin) and on average not statistically different (unpaired Student’s *t*-test, *p* = 0.998, *n* = 13). In a second experiment, EGFP-expressing cells were virally transduced to express tdTomato (Figure 3B). Images of astrocytes expressing both EGFP and tdTomato were taken, and the VFs of astrocytes were measured from both channels using identical ROIs. Again, the VFs obtained from both channels were almost identical (Figure 3B). These experiments demonstrate that VF measurements from astrocytes are independent of the used dye, the method for dye delivery and of the dye concentration because expression levels of tdTomato and EGFP can vary considerably between cells. We next compared VFs of astrocytes from acute slices to VFs recorded from *in vivo* data, because astrocytes in acute slices had been suggested to rapidly show a reactive phenotype accompanied by a significant loss of small astrocyte processes (Takano et al., 2014). We compared VFs of EGFP-expressing astrocytes in the somatosensory cortex (layer 2/3) in acute horizontal slices and in anesthetized mice (Figure 3C, left and middle panel, respectively). No statistically significant difference between the VFs in the two preparations was detected (Figure 3C, right panel). Note that in these experiments the analysis ROI included the soma and the perisomatic region. This indicates that there is no major loss of astrocyte volume across the astrocyte territory in this slice preparation and that VF measurements can be comparable between microscopes and preparations. Finally, VF measurements do not need to cover the entire astrocyte. Instead, it might be of interest how specific subregions of the astrocyte change or how the astrocyte volume is distributed across the cell’s territory. An example of such a refined analysis is shown in Figure 3D. Here, the entire astrocyte is divided into a grid of quadratic ROIs (Figure 3D, left panel), for each the VF is determined and the probability distribution of VFs across the astrocyte territories is calculated (Figure 3D, middle panel). As expected, low local VFs below ~0.2, which represent parts of the astrocyte territory dominated by small astrocytic processes, are most frequent. Across all cells, 96% of all ROIs have a local VF of less than 20%. In contrast, high local VFs, i.e., ROIs being more extensively filled by larger branches, are far less often encountered. This type of analysis can be useful to reveal, for instance, in which part of the astrocyte morphology is changing (Henneberger et al., 2020), and to correlate subcellular structures with properties of astrocytic Ca^2+^ signals (King et al., 2020). In addition, we performed a Sholl-like analysis and plotted the average VF and the coefficient of variation of the VF against the distance from the soma center (Figure 3D, right panel). The VF drops sharply at 3–5 μm from the soma center to average values below 10%, illustrating that even at short distances from the soma the territory of the astrocyte is predominantly filled by small astrocyte processes. At the transition zone, the coefficient of variation is highest because ROIs with high VF, i.e., ROIs covering major branches are mixed with low VF ROIs.

### Astrocytic Volume Fraction and Segment Measurements in Three Dimensions

All analyses above were performed on single optical sections through the soma of astrocytes. However, astrocytes display a complex three-dimensional morphology. We, therefore, tested to what extent single-plane volume fractions and segment counts are representative of the entire cell. This was done by recording imaging stacks of single astrocytes and then obtaining three two-dimensional optical sections through the soma from each stack (X-Y, X-Z, Y-Z). See Figure 4A for an example. First, we calculated the VF in each set of optical sections as described above and correlated the results between the three orientations. As displayed in Figure 4B, the VFs are highly correlated between the different orientations. Second, we determined how many astrocytic segments can be detected in the three corresponding optical sections. In this analysis, the number of detected astrocytic segments were divided by the area of the analysis ROI to obtain the segment density (Figure 4C). This was done for comparisons because the sizes of analysis ROIs vary between orientations. The segment density was also highly correlated between planes although it is noteworthy that the segment density in sections along the Z-axis is generally lower when compared to the X-Y plane. This is expected because both the nominal resolution in our experiments and, more importantly, the optical resolution is significantly lower in the Z-direction compared to X-Y (Helmchen and Denk, 2005; Yang and Yuste, 2017), which can be easily spotted by comparing the middle and right panel of Figure 4A with the left panel. For all statistical details please see the legend of Figure 4. There are two important conclusions that can be drawn from these observations. On one hand, it is convenient for the experimenter that a single focal plane is representative of the entire astrocyte. On the other hand, our data reveal that volume fraction and segment density are set to some extent on the level of individual cells, because they are similar across different sections through the same cell, even though they vary considerably between cells.

### Heterogeneity and Development of Astrocyte Morphology

**Figure 5:**
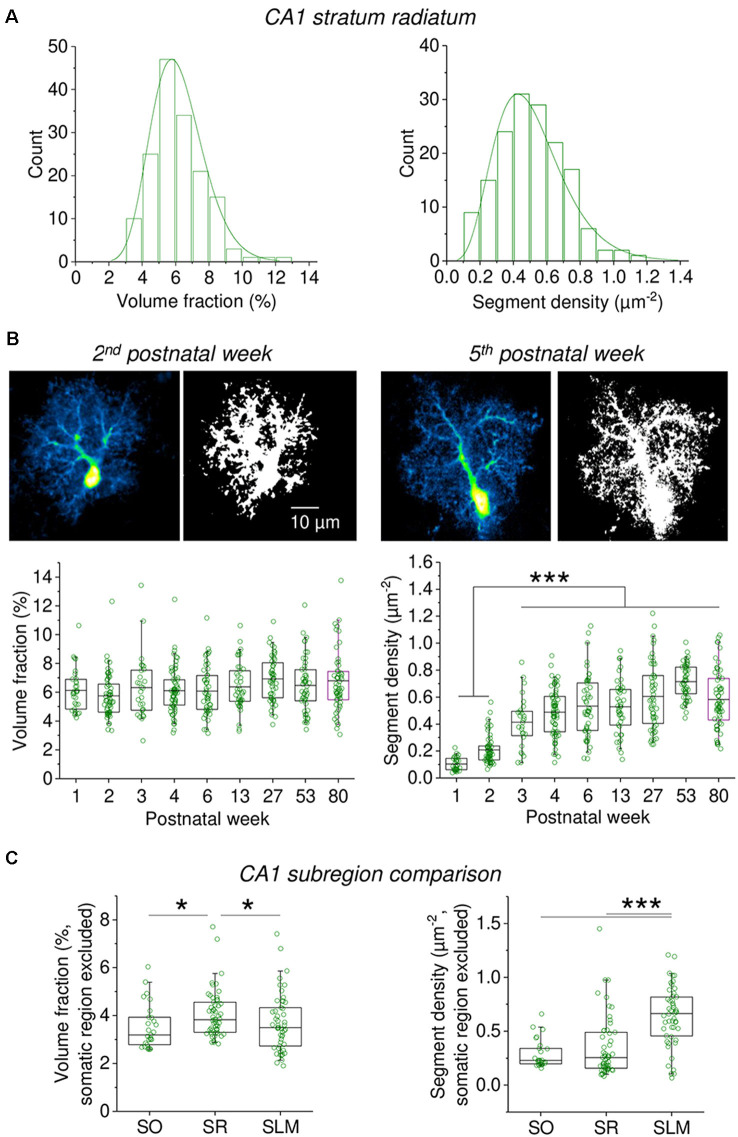
Capturing the heterogeneity and developmental profile of astrocyte morphology using measurements of volume fraction and segment density. **(A)** The volume fraction and segment density of EGFP-expressing astrocytes in the CA1 stratum radiatum was analyzed in acute slices (*n* = 158). Somatic reference region as before. Entire cross-sections including the soma were analyzed (see Figure 3). A significant heterogeneity of both parameters was observed (coefficient of variation of volume fraction = 0.26; of segment density = 0.39). **(B)** Developmental changes of volume fraction and segment density (*n* = 437 astrocytes). Time windows in postnatal weeks and number of astrocytes from left to right: 1st *n* = 31, 2nd *n* = 59, 3rd *n* = 28, 4th *n* = 67, 5th–7th *n* = 49, 12th–14th *n* = 42, 26th–28th *n* = 52, 52nd–54th *n* = 50, 79th–81st *n* = 59). Like in **(A)**, entire cross-sections of astrocytes including the soma were analyzed (somatic reference region). Examples of astrocytes from the 2nd and 5th postnatal week are shown (top panel pairs, scale bar applies to all panels). Each image pair is a representative astrocyte shown before (left) and after (right) binarization for quantifying astrocyte segment density. Note the more granular binarized image for the astrocyte from a 5-week-old animal. See Supplementary Figure 2 for further examples. Lower panels illustrate the developmental profile. Left: volume fraction (one-way ANOVA *p* = 0.01846; Tukey *post hoc* *p* < 0.05 for week 2 vs. 27 and week 2 vs. 80). Right: segment density (one ANOVA *p* < 0.0001; Tukey *post hoc* *p* < 0.0001 for all combinations of weeks 2 and 3 vs. weeks 3–80). Individual circles represent measurements from single astrocytes. Box represents mean and 25th and 75th percentile. Whiskers represent 5th and 95th percentiles. **(C)** The volume fraction and segment density were measured in cross-sections of astrocytes from the *stratum oriens* (SO, *n* = 27), *stratum radiatum* (SR, *n* = 55) and *stratum lacunosum-moleculare* (SLM, *n* = 50). A total of six mice 6–8-week-old were used. Both parameters were analyzed in regions of interest sparing the somatic region (see Figure 2A). Somatic reference region as elsewhere. Left panel: volume fraction comparison (Kruskal Wallis *p* = 0.0110, Dunn *post hoc* *p* < 0.05 for SR vs. SO and SLM). Right panel: segment density comparison (Kruskal Wallis *p* < 0.0001; Dunn *post hoc* *p* < 0.0001 for SLM vs. SO and SR). *p < 0.05 and ***p < 0.001.

The rapidness and relative ease with which the information about fine astrocyte processes can be captured using VF and segment density measurements allows the experimenter to study larger populations of astrocytes. We first investigated the heterogeneity among 158 astrocytes in the stratum radiatum of the hippocampus (CA1) and found that both the astrocyte VF and segment density are highly variable with coefficients of variation of 0.26 and 0.39, respectively (Figure 5A). Interestingly, there is no correlation between the VF and segment density of individual astrocytes (Spearman’s rank correlation, *R* = 0.013, *p* = 0.878). Also, see Supplementary Figure 1. Building on this observation we next studied a total of 437 astrocytes (including the 158 astrocytes from above) in acute hippocampal slices prepared from animals with postnatal ages of 1 week to 80 (Figure 5B). When the VF and segment density of these astrocytes were analyzed, we observed that the average VF was surprisingly constant throughout the life of mice whereas the segment density increased during the first three postnatal weeks and then remained stable overall. The gradual appearance of a higher astrocytic segment density, i.e., of a more strongly ramified astrocyte morphology and a stable VF suggests that the astrocyte process undergo a developmental transition during which finer astrocyte processes are being generated at the expense of their volume. Finally, we compared the VF and segment density of astrocytes between different layers of the hippocampal CA1 region (Figure 5C). While the VF of astrocytes in the *stratum radiatum* (SR) was higher than in *stratum lacunosum-moleculare* (SLM) and *stratum oriens* (SO), the segment density of astrocytes in the SLM was higher when compared to the SR and SO. This suggests that astrocytes in the SLM have a more ramified morphology with more fine processes, which overall have a smaller volume compared to SO and SR astrocytes.

**Figure 6:**
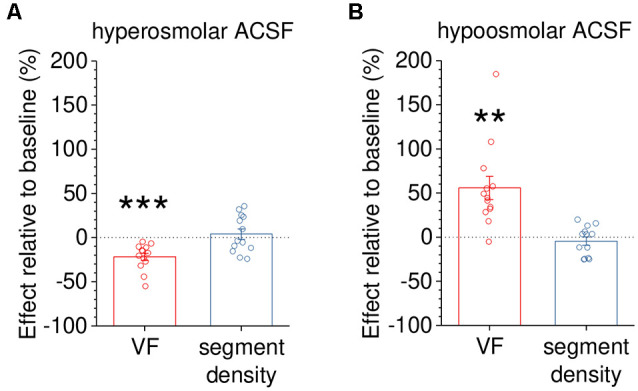
Capturing dynamic changes of astrocyte volume in response to altered extracellular osmolarity. To demonstrate that volume fraction (VF) measurements are a sensitive indicator of volume changes of small astrocyte processes, astrocytes were acutely exposed to hyper- or hypoosmolar extracellular solution (see “Materials and Methods” section). The morphology of EGFP-expressing astrocytes (not illustrated) was analyzed before (baseline) and 10 min after changing the extracellular solution. Changes in VF and segment density were expressed as per cent change relative to the baseline recording. **(A)** The VF was significantly reduced by application of hyperosmolar solution (~400 mOsm/kg, −21.8 ± 4.1%, *p* < 0.001) whereas the segment density was not (+4.0 ± 5.8%, *p* = 0.510, *n* = 13 and paired Student’s *t*-test for both tests). **(B)** In a second set of experiments, the VF of astrocyte processes was increased by exposure to hypoosmolar extracellular solution (~200 mOsm/kg, +55.8 ± 13.2%, *p* = 0.00117) whereas the segment density was not affected (−4.5 ± 4.6%, *p* = 0.351, *n* = 13 and paired Student’s *t*-test for both tests). Overall, changes of VF and segment density did not display a statistically significant correlation (Spearman’s rank correlation, *R* = −0.246, *p* = 0.226, *n* = 26). Also, see Supplementary Figure 1C. **p < 0.01 and ***p < 0.001.

In a final set of experiments, we tested how measurements of VF and segment density change in response to rapid volume changes of peripheral astrocytic processes. The latter were induced by acutely reducing or increasing the osmolarity of the extracellular solution. The prediction is that the expected volume increase in hypoosmolar solution would increase the VF and the volume decrease in hyperosmolar solution would decrease the VF, respectively. This was indeed observed for the VF (Figure 6) whereas no statistically significant effect on the segment density was detected (see figure legend for statistical details).

## Discussion

The morphology of astrocytes and its changes can be characterized on many levels: from the volume of their territory and number of main branches to the precise geometry of their small perisynaptic processes. Here, we describe two measures of astrocyte morphology, the astrocytic volume fraction and the segment density, that can be rapidly extracted from a single optical section through an astrocyte and its cells body. While the volume fraction is directly related to the number of astrocytic processes and their average volume in the optical section/region of interest, the segment density is representative of but not equal to the number of astrocyte processes (also see below). For the interpretation of both parameters, there are several important points to consider. Also, the entire approach was designed with three-dimensional astrocytes in organized tissue such as brain slices or *in vivo* experiments in mind and requires modification for flat preparations.

### Volume Fraction

Determining the volume fraction requires a reference, where 100% of the optical section is filled by the astrocyte. This is the case for the astrocyte soma with diameters of ~ 5–10 μm and the typical point spread function (PSF) of the two-photon excitation microscopes used in this study (full width at half-maximum, 0.35–0.50 μm in x-y and 1.5–2.5 μm in z), because the PSF is fully within the cell at the somatic region (Savtchenko et al., 2018; Henneberger et al., 2020). This would need to be verified for other microscopes or techniques. In addition, the z-positions of optical sections through the soma need to be carefully set and maintained, e.g., for time-lapse imaging. The second assumption of VF measurements is that the cytosolic concentration of the dye is the same throughout the cell. Aggregation of fluorescent proteins, their accumulation in cellular sub-compartments, or rapid dye bleaching can be incompatible with reliably measuring the volume fraction. Special care needs to be taken when astrocytes are acutely filled with a dye, for instance *via* a patch pipette. In this case, sufficient access to the cell and stable filling needs to be ensured. Filling astrocytes with dyes with a low molecular weight represents the additional challenge that these dyes can escape from the patched cell through gap junction and hemichannels, which could lead to intracellular concentration gradients. Sufficiently large dextran-conjugated dyes can be used as an alternative (Figure 3A; Breithausen et al., 2020) or appropriate control experiments need to be performed (Henneberger et al., 2020). However, larger dyes re-equilibrate more slowly across the cytosol after a morphology change and therefore report these changes less rapidly (Henneberger et al., 2020). Finally, basic requirements of quantitative microscopy need to be met, i.e., imaging settings must be such that there is no clipping of the fluorescence intensity distribution (e.g., no saturation) and that there is a linear relationship between dye concentration in an imaged volume and fluorescence intensity. For interpreting the volume fraction and its changes, it needs to be kept in mind that it is the product of the number of processes and their average size, and therefore cannot distinguish between the two factors on its own. Also, it captures the cytosolic volume and may therefore be insensitive to changes or redistribution of intracellular organelles. Despite these potential drawbacks, our current study demonstrates that the astrocytic volume fraction can be easily and reliably obtained across various experimental approaches. In addition, we have verified it previously using electron microscopy (Medvedev et al., 2014), and successfully used astrocytic volume fraction measurements to constrain the morphology of realistic *in silico* models of astrocytes (Savtchenko et al., 2018), to correlate astrocyte morphology and Ca^2+^ signaling (King et al., 2020) and to demonstrate astrocyte process withdrawal from synapses after induction of synaptic long-term potentiation (Henneberger et al., 2020).

### Segment Density

To complement volume fraction measurements, we have established the segment density as a second parameter for describing the abundance of small astrocyte processes. Because diffraction-limited microscopy does not fully resolve individual processes, the calculated segment density is not the true density of astrocyte cross-sections in an optical section. However, our simulations, emulations of diffraction-limited microscopy on super-resolved ExM data and analysis of pre- and post-expansion data showed that the detected segment density is lower but strongly correlated with the true value. It is intuitive that the quantified segment density depends on the dimensions of the microscope’s PSF. Therefore, absolute values of segment density may not be comparable between microscopes. Indeed, it is our experience that all imaging settings (e.g., objective, excitation wavelength, fluorescence detection) should be kept as constant as possible to compare individual recordings of segment density. Such considerations play a lesser role in experiments with online monitoring of astrocyte morphology and acute manipulations, where it is usually easier to keep imaging conditions constant. Increases or decreases of the segment density, or differences between two experimental groups, indicate a different number of astrocytic process cross-sections in the optical sections. For example, an increase in the astrocytic segment density could reflect the outgrowth/appearance of new astrocytic processes. Such observations can be easier to interpret when combined with volume fraction measurements. For instance, the appearance of new astrocyte processes with comparable size should also increase the volume fraction. Similarly, the disappearance of astrocyte processes or a less ramified morphology can reduce the segment density and the volume fraction. In contrast, a change of volume fraction but not the segment density would indicate that primarily the volume of astrocyte processes is affected. It should be noted, however, that an isolated change of process volume could also affect the detection of astrocytic processes. For example, swollen processes could be more difficult to separate in diffraction-limited microscopy leading to fewer detected segments and thus a lower segment density (but see Figure 6). Whether such effects play a role depends on the optical resolution of the used microscope.

Interestingly, we found no correlation between the volume fraction and segment density of individual astrocytes when we studied sufficiently large populations (Supplementary Figure 1). This indicates, for example, that an astrocyte with a high density of small processes does not simply arise from an astrocyte with a low density of processes by the addition of new processes of the same volume, because that would also increase the volume fraction. Instead, our data imply that an increase in process density affects the average size of astrocytic processes. The development of astrocyte morphology could be an example of such a process (see below). Studying such relationships in further detail will require isolated manipulation of either astrocyte process volume or density.

### Selecting the Region of Analysis

In this study, we mostly selected the region for analysis manually. Naturally, the calculated volume fraction and segment density will depend on how this region was chosen. If it includes the soma, for instance, the volume fraction across the region of analysis will be higher compared to an analysis region sparing the soma. Similarly, volume fraction and segment density will change if blood vessels are not excluded or endfeet are included in the analysis. Many alternatives to manual selection are possible. For instance, multiple regions of interest covering the entire astrocyte can be defined automatically (Figure 2; King et al., 2020), a standardized region of interest in the periphery of astrocytes can be defined (Henneberger et al., 2020) or individual spot-like regions of analysis can be placed at a site of experimental manipulations, e.g., glutamate uncaging (Henneberger et al., 2020). Automated detection of astrocyte boundaries would be helpful for the analysis of large populations of astrocytes. This will require images in which the outer boundaries of single astrocytes can be reliably traced automatically, which could be the case for animal models with consistently sparse or combinatorial expression of fluorescent reporters in astrocytes (Livet et al., 2007; García-Marqués and López-Mascaraque, 2013).

### Capturing Heterogeneity and Developmental Changes of Astrocyte Morphology

After testing the robustness of volume fraction and segment density measurements in various experimental approaches in acute slices and *in vivo*, we used both parameters to explore the heterogeneity and development of astrocyte morphology. Interestingly, we observed that within their territories the astrocyte volume fraction remained constant while the segment density increased over the first three postnatal weeks. This indicates that astrocyte ramification increases over this period of development and that small astrocyte processes become more abundant but also thinner, which is similar to a previous qualitative assessment of astrocyte development (Bushong et al., 2004). The advantage of easily obtainable measures of morphology over a qualitative assessment is that it can enable the experimenter to quantify the contribution of specific signaling molecules, such as GTPases of the Rho family (Zeug et al., 2018; Müller et al., 2021) or BDNF (Holt et al., 2019), to the development and heterogeneity of astrocyte morphology. Because the astrocyte volume fraction and segment density can be quickly extracted from a single optical section through a dye-filled astrocyte and its soma, and because this is representative of the entire astrocyte, this approach allowed us to analyze hundreds of astrocytes using two-photon excitation fluorescence microscopy in acute slices and *in vivo*. This approach should also be useful in other preparations, in fixed tissue and for microscopy methods such as confocal microscopy, if the aforementioned conditions are met. Despite these advantages, our approach cannot replace the methods that accurately quantify the geometry and branching patterns of large astrocyte processes, see for instance Tavares et al. (2017), or that resolve the fine geometric details of astrocyte structure such as electron (Ventura and Harris, 1999), STED (Arizono et al., 2020, 2021) or expansion microscopy (Herde et al., 2020), among other techniques as recently reviewed (Heller and Rusakov, 2017). However, the approach can be used to efficiently screen for biologically relevant scenarios by comparing large populations of astrocytes in different conditions or by online monitoring of astrocyte morphology changes, their triggers and functional consequences. Interesting scenarios can then be explored using the more complex methods mentioned above. Our recent study on synaptic plasticity is one example (Henneberger et al., 2020), in which results from volume fraction measurements were then further investigated using STED and electron microscopy. Other options are techniques for locating neuron-astrocyte interaction sites using proximity assays (Octeau et al., 2018) and for identifying the proteins enriched at such contact sites (Takano et al., 2020).

## Data Availability Statement

The raw data supporting the conclusions of this article will be made available by the authors, without undue reservation.

## Ethics Statement

The animal study was reviewed and approved and all animal procedures were conducted in accordance with the regulations of the European Commission and all relevant national and institutional guidelines and requirements. All procedures have been approved by the Landesamt für Natur, Umwelt und Verbraucherschutz Nordrhein-Westfalen (LANUV, Germany) where required.

## Author Contributions

DM, CD, PU, AP, SA, MH, CB, and CH performed and analyzed experiments in acute slices and expansion microscopy. PU, AD, GP, and CH performed and analyzed *in vivo* experiments. PG and SS designed and produced AAVs. CH conceived the study, performed the modeling, and wrote the manuscript. All authors contributed to the article and approved the submitted version.

## Conflict of Interest

The authors declare that the research was conducted in the absence of any commercial or financial relationships that could be construed as a potential conflict of interest.
