# Supplementary material for: Heterogeneity and Development of Fine Astrocyte Morphology Captured by Diffraction-Limited Microscopy
Source: Front Cell Neurosci. 2021 Jun 4;15:669280. doi: 10.3389/fncel.2021.669280 (PMC8211899; doi:10.3389/fncel.2021.669280)
Supplement: Supplementary file 1 [file Image_1.pdf]

Supplementary figure 1

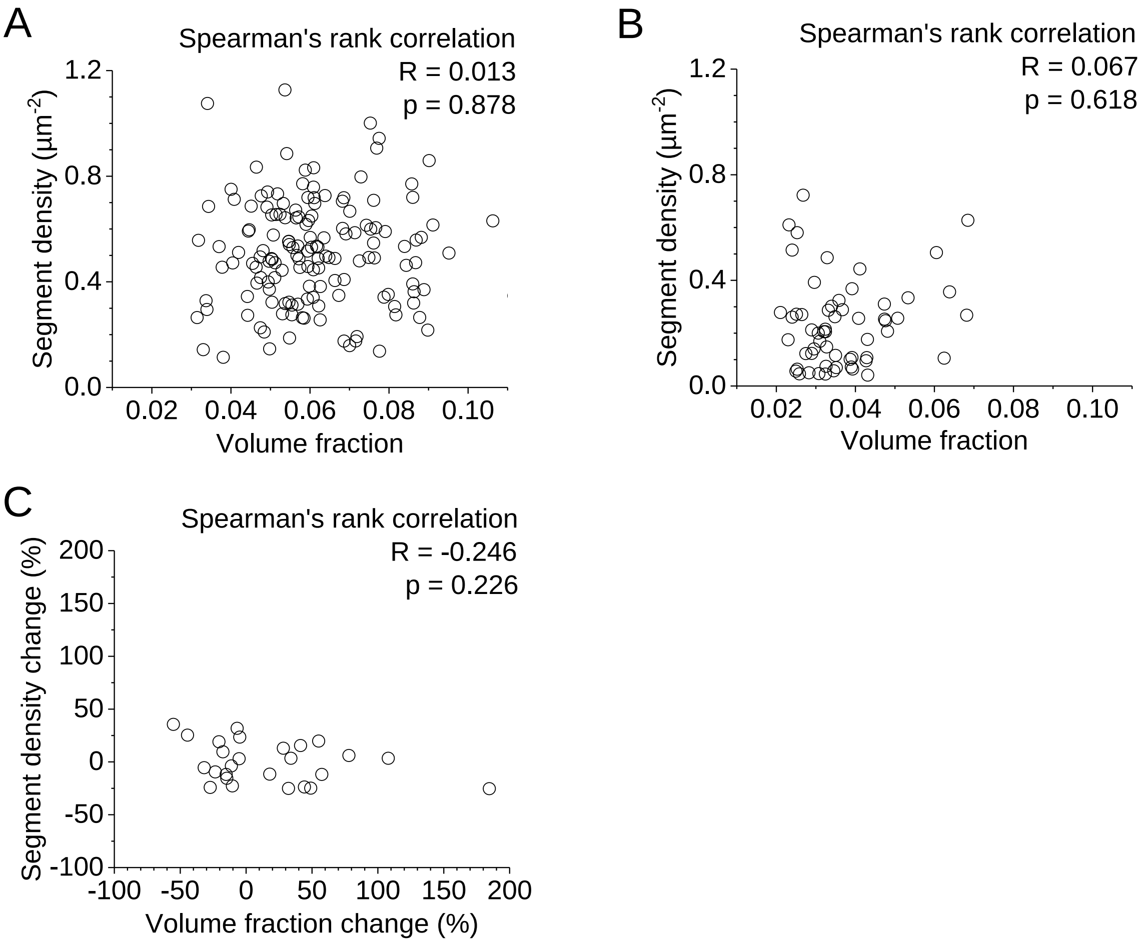

Supplementary figure 1: No correlation between volume fraction and segment density of individual astrocytes across populations. A) Comparison of volume fractions and segment densities measured from 158 astrocytes. Same data as in **Fig. 5A**. No statistically relevant correlation was found across the population. **B)** Comparison of volume fractions and segment densities recorded from 58 astrocytes. Same data as in **Fig. 4**. Note that this data set was obtained using a different microscope than in **A**. Therefore, absolute segment densities differ between the panels. Also, parts of the cell containing major astrocyte processes were excluded from this analysis, which leads to an overall lower volume fraction. See legend of **Fig. 4**. No statistically relevant correlation was found across the population of astrocytes. **C)** Comparison of volume fraction and segment density changes in experiments with hypo- and hyperosmolar solution from **Fig. 6**. No statistically relevant correlation was found.

Supplementary figure 2

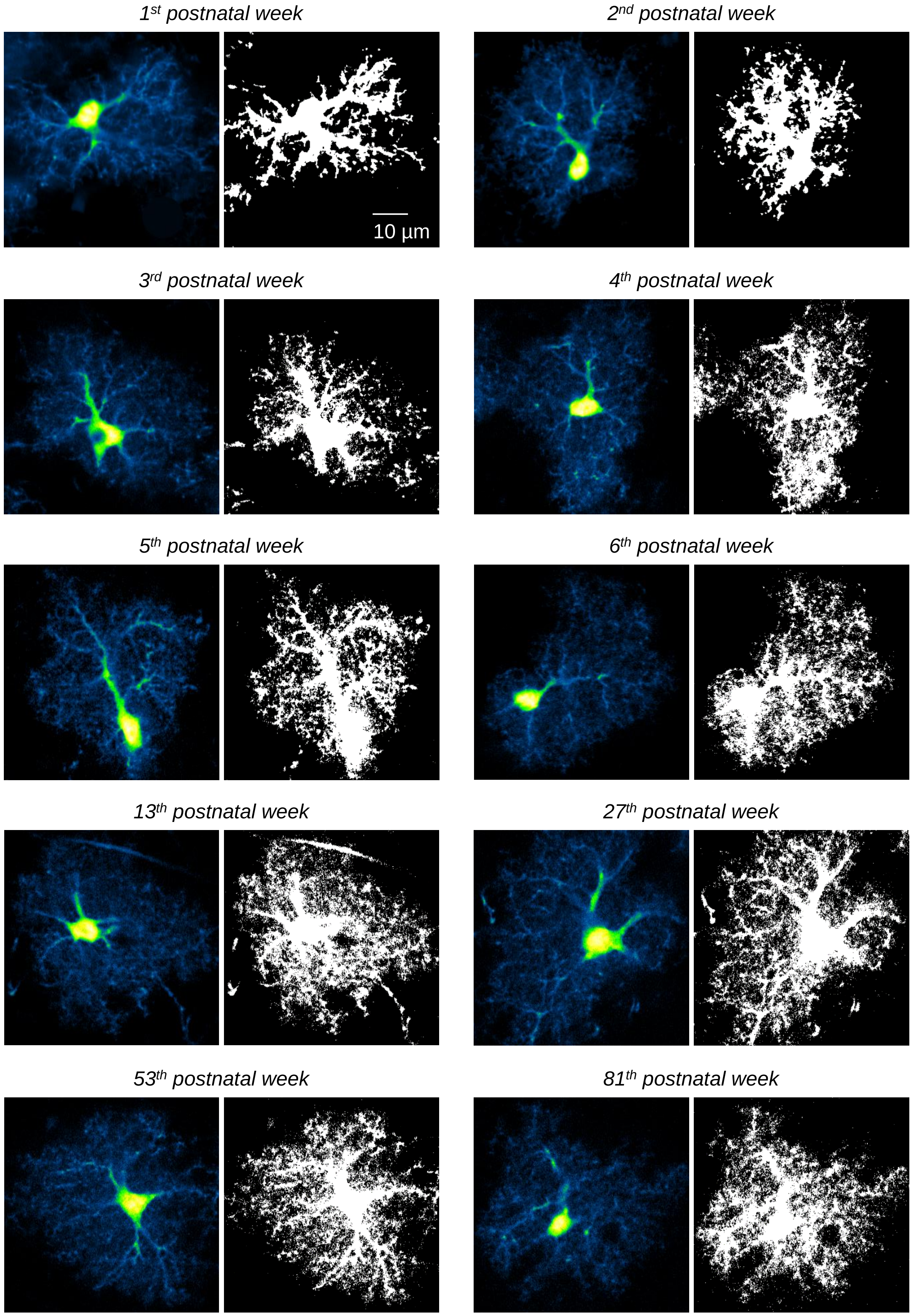

Supplementary figure 2: **Additional examples for Fig. 5.** Each pair of images represents a single astrocyte and the corresponding binarized image. Note how smaller structures become more prominent over the 1st four weeks of postnatal development. The scale bar in

the top image applies to all image panels. For further details, please see Fig. 5 and the main text.
